# Cerebellar radiological abnormalities in children with neurofibromatosis type 1: part 1 - clinical and neuroimaging findings

**DOI:** 10.1186/s40673-018-0093-y

**Published:** 2018-11-01

**Authors:** Michael S Salman, Shakhawat Hossain, Lina Alqublan, Martin Bunge, Katya Rozovsky

**Affiliations:** 1 Section of Pediatric Neurology, Winnipeg Children’s Hospital and Department of Pediatrics and Child Health, Max Rady College of Medicine, Rady Faculty of Health Sciences, University of Manitoba, AE 308, 820 Sherbrook Street, Winnipeg, MB R3A 1R9 Canada; 2 Department of Mathematics and Statistics, University of Winnipeg, Winnipeg, MB Canada; 3 Department of Radiology, Max Rady College of Medicine, Rady Faculty of Health Sciences, University of Manitoba, Winnipeg, MB Canada; 4 Present Address: Department of Radiology, King Fahad Armed Forces Hospital, Jeddah, Western region Saudi Arabia; 5 Section of Pediatric Radiology, Department of Radiology, Max Rady College of Medicine, Rady Faculty of Health Sciences, University of Manitoba, Winnipeg, MB Canada

**Keywords:** Cerebellum, Neurofibromatosis type 1, Neuroimaging, Pediatrics, Clinical features

## Abstract

**Background:**

Many children with neurofibromatosis type 1 (NF1) have focal abnormal signal intensities (FASI) on brain MRI, whose full clinical impact and natural history have not been studied systematically. Our aims are to describe the clinical and neuroradiological features in children with NF1 and cerebellar FASI, and report on the natural history of FASI that display atypical features such as enhancement and mass effect.

**Method:**

A retrospective review of the hospital charts and brain MRIs was performed on children from Manitoba diagnosed between 1999 and 2008 with NF1, who also had cerebellar FASI on MRI.

**Results:**

Fifty patients (mean age: 16.1y, minimum-maximum: 6.4 - 30y, 27 M) were identified. Mean duration of follow up was 10.1y. Developmental delay, learning disabilities, tumors, and visual signs occurred commonly. Cerebellar signs were not reported. Mean age of the patients at baseline MRI was 7.8 (SD: 4.5) years. FASI occurred in several brain locations and were rarely confined to the cerebellum. FASI displayed mass effect and enhancement infrequently but were associated with malignancy only once. The number of FASI at baseline MRI was significantly less in patients with attention deficient hyperactivity disorder and more if a first degree relative had NF1 or if they had decreased visual acuity.

**Discussion:**

Patients with NF1 and cerebellar FASI do not have motor or consistent non-motor (e.g. developmental delay or learning disabilities) cerebellar features. The number of FASI may correlate with some clinical features. FASI may display enhancement and mass effect but they rarely become malignant.

## Background

Neurofibromatosis type 1 (NF1) is an autosomal dominant neurocutaneous disorder with an estimated incidence of 1/3500 live births [1]. Roughly half of all cases are inherited, while the other half are due to de novo mutations. Diagnosis requires the presence of at least two of seven major criteria: six café-au-lait spots, axillary or inguinal freckling, two neurofibromas or one plexiform neurofibroma, two Lisch nodules, an optic glioma, a distinctive osseous lesion, or a first-degree relative with NF1. These features tend to manifest in a characteristic sequence throughout infancy, childhood and adolescence [1]. NF1 exhibits complete penetrance but extremely variable expressivity [2]. This, combined with the infrequent correlation between specific genetic mutations and clinical phenotype [2, 3], make it difficult to predict the severity of NF1 for a given patient.

Patients with NF1 are at increased risk of developing both benign and malignant tumors compared to those in the general population [4]. The most common intracranial neoplasms in patients with NF1 are optic pathways gliomas followed by low-grade astrocytomas of the posterior fossa [5–7]. These tumors are usually less aggressive than comparable lesions in children without NF1 [6, 7]. Tumors of the optic pathways may present in early childhood with vision loss, proptosis or precocious puberty [1, 2, 7]. However, many are asymptomatic and are discovered incidentally on MRI, as are the majority of tumors in the posterior fossa with rare exceptions [5, 6, 8]. Screening for these tumors in asymptomatic individuals is controversial [5, 7].

Patients with NF1 may have behavioral impairment and cognitive dysfunction. While many studies have been done on the subject, results are often conflicting and no clear NF1 cognitive profile has emerged. Visuospatial and fine motor deficits have long been considered hallmark neurological features of NF1 [9, 10]. Multiple studies have reported that children with NF1 have normal intellectual ability, but as a group tend to score below-average on IQ tests when compared to controls [9–11]. These children often have widespread academic difficulties and it is estimated that between 35 and 65% have a learning disability [9]. In addition, half of them meet the criteria for attention-deficit hyperactivity disorder [9].

Many attempts have been made to correlate these cognitive and neurological deficits with radiologic findings. The most common abnormalities found on brain MRI are regions of increased signal visible on T2-weighted images i.e., focal abnormal signal intensities (FASI). These are believed to be present in 60–80% of pediatric patients with NF1 [12], though some studies have reported a prevalence as high as 93% [5, 13]. FASI are most common in the basal ganglia, but are also found in the thalamus, brainstem, cerebellum and occasionally cerebral hemispheres [13–15]. They do not typically exhibit mass effect or enhancement, and are not associated with focal neurological defects [10, 13]. Studies examining the clinical significance of FASI have contradictory findings, though most agree that there is no correlation between neurological deficits and the presence or absence of FASI [16]. There is evidence to suggest that their location, but not their number or size, may have neuropsychological correlates. Specifically, FASI located in the thalamus are associated with cognitive impairment [11, 12]. Despite being poorly understood, there are some who feel that FASI in children are pathognomonic of NF1 and recommend their presence be incorporated as one of the major diagnostic criteria of the disease [17].

The cerebellum plays an important role in the processing of non-motor in addition to motor tasks. Many studies support non-motor roles for the cerebellum in cognition [18]. Children with cerebellar disorders have been documented to have cognitive and neuropsychiatric disorders. In addition, developmental delay, learning disabilities, and behavioral problems have been commonly reported in children with developmental cerebellar disorders [18]. Therefore, we hypothesized that children with cerebellar FASI were likely to have cerebellar motor signs and developmental delay and/ or learning disabilities.

The aims of this study are to: Describe the clinical features in children with NF1 and cerebellar FASI on MRI, correlate the clinical features, especially developmental delay, learning disabilities, and examination findings, with baseline neuroradiological abnormalities, and describe the natural history of FASI that display atypical features including mass effect and enhancement.

## Methods

Electronic MRI neuroimaging reports stored in the radiology information system at our Health Sciences Centre (HSC) for the 10-year period starting January 1, 1999 were read and flagged for the occurrence of posterior fossa abnormalities. Patients with NF1 who had cerebellar radiological abnormalities mentioned on any brain MRI report when they were less than 17 years old between 1999 and 2008, were selected. All patients were seen in Winnipeg Children’s Hospital for a clinical evaluation. Patients from neighboring provinces who attended Winnipeg Children’s Hospital were also included. Ethical approval for the study was given by the Research Ethics Board of the University of Manitoba.

Inclusion criteria for the study were: (1) Patients were less than 17 years old during the study period, (2) patients had NF1, and (3) patients had cerebellar abnormalities on MRI. Exclusion criteria were: (1) Patients with solid central nervous system tumors unrelated to NF1, (2) trauma involving the posterior fossa, (3) significant brain malformations and anomalies unrelated to NF1, (4) patients who had brain radiotherapy or chemotherapy before their first brain MRI, and (5) patients with other unrelated brain disease e.g. demyelinating disorders.

The hospital chart, pediatric neurology clinic letters, and genetic clinic letters were reviewed in each of these patients. The relevant data available until 2013 were extracted and entered into a purpose-specific database. The data consisted of demographic information, duration of follow-up period, pregnancy, birth and perinatal history, family history of NF1, presenting symptoms, presence of features seen in NF1 including café-au-lait spots, axillary freckling, neurofibromas, bone dysplasia, NF1 related tumors; other diagnoses, developmental milestones, learning disabilities, and physical exam findings especially ocular and central nervous system examination abnormalities.

Brain MRI was acquired on 1.5- or 3-Tesla MRI scanner (GE) using standardized protocol with sagittal T1-weighted, axial/ coronal T2-weighted, and axial/ coronal fluid-attenuated inversion recovery (FLAIR) images. Supplemental imaging sequences were performed as needed including T2*, DWI, ADC (apparent diffusion coefficients) maps, fast spoiled gradient echo (FSPGR) images, and MRA. Contrast with Gadolinium was given at the discretion of the radiologist. All initial and subsequent brain MRI images available that were completed by June 2014 were reviewed independently by two pediatric radiologists with expertise in neuroimaging. Disagreements were resolved by consensus.

Age at the time of the MRI scan, structural and signal abnormalities in the cerebellar vermis, cerebellar hemispheres, brainstem, and supratentorial structures including the cortex and white matter, basal ganglia, thalami, hypothalamus, and optic nerves/ chiasm were recorded, as well as the presence of cerebellar hypoplasia (small size but normal shape) or cerebellar atrophy (shrunken size with prominence of the cerebellar folia). Optic nerve(s) and/ or chiasm gliomas (optic pathways gliomas) were diagnosed when these structures were enlarged/ thickened on MRI. Detailed information was collected on FASI on FLAIR images including their number, locations, diameter (defined as a straight line passing from side to side through their center and thus representing their maximum length), signal characteristics, uptake of contrast on T1-weighted images, and mass effect on adjacent structures. Brain locations were divided as follows: cerebellum, brainstem (midbrain, pons, medulla, cerebellar and cerebral peduncles), thalamus/ hypothalamus, basal ganglia/ internal capsule (these structures were combined since lesions in the internal capsules tended to involve the basal ganglia and it was difficult to separate the two, as was also reported previously) [19], and cerebrum (cortex, subcortical and periventricular white matter, hippocampus, corpus callosum, and fornix).

The data were converted to numerical variables using a numerical coding scheme to render the data suitable for statistical analysis and modelling. Mean and median were used to describe the normally distributed and skewed data, respectively.

## Results

Fifty patients fulfilling the inclusion criteria were identified. Their mean age at the end of the study period was 16.1 years (minimum: 6.4, maximum: 30 years). There were 27 males and 23 females. Mean duration of follow up was 10.1 years. Table 1 shows further demographic information. There were four deaths, all caused by malignant tumors (Table 2). Table 3 shows selected information on maternal health, pregnancy and delivery. Most patients were born near or at term and no patient was born before 33 weeks gestation. The presence of café au lait macules and a positive family history of NF1 were the most common reasons for the initial assessments of these patients (Table 4). Table 5 shows the clinical features in our cohort. Tumors, developmental delay, and learning disabilities occurred commonly. Eye movement exam was documented poorly. Saccadic smooth pursuit was reported in four patients. Cerebellar motor signs were not reported including head titubation, dysarthria, dysmetria, dysdiadochokinesia, intention tremor, rebound, gait ataxia, and wide-base gait. None of the patients had dyskinesia.

**Table 1:** Demographics of NF1 patients

	Details
Total number of patients	50
Gender M/F	27/23
Mean age [SD] at end of study in years (y)	16.13 [5.5]
Median age [min.-max.] at symptom/ sign onset (y)	0.4 [0.00–8.5]
Median age [min.-max.] at first clinical assessment (y)	1.9 [0.3–16.3]
Median age [min.-max.] at diagnosis of NF1 (y)	2.2 [0.00–11.3]
Mean age [SD] at last clinical assessment (y)	13.6 [6.4]
Mean duration [SD] of clinical follow up (y)	10.1 [5.9]
Total number of deaths	4
Median age [min.-max.] at death (y)	18.2 [2.3–28.1]
Median maternal age {25th–75th %} and [min.-max.] at birth (y)	27 {21–32}, [17–52]

*min*. minimum, *max*. maximum

**Table 2:** Mortality in NF1 patients

Age at death (y)	Sex	Diagnosis
2.3	M	High grade pilomyxoid astrocytoma of the hypothalamus presenting with symptoms and signs of raised intracranial pressure
13.0	M	Disseminated metastatic high grade undifferentiated sarcoma with neuronal phenotype presenting with spinal cord compression
23.4	F	High grade malignant peripheral nerve sheath tumor presenting with cauda equina syndrome
28.1	M	Metastatic high grade spindle cell sarcoma arising from the pelvis and presenting with hematuria and bilateral hydronephrosis

**Table 3:** Maternal health, pregnancy and labor for NF1 patients

	Number of patients (%)	^a^ Total number of patients
Maternal health prior to pregnancy		
NF1	7 (20.6)	34
History of spontaneous fetal loss	9 (18)	50
Gestational hypertension	7 (18.4)	38
Fetal exposures during pregnancy		
Smoking	9 (33.3)	27
Alcohol	6 (22.2)	27
Marijuana	4 (16.7)	24
Radiation	1 (12.5)	8
Delivery		42
Uncomplicated SVD	32 (76.2)	
^b^ Complicated SVD	3 (7.1)	
Planned C/S	5 (11.9)	
Emergency C/S	2 (4.8)	
Gestational age in weeks		42
Term (≥ 37 weeks)	36 (85.7)	
36 weeks	5 (11.9)	
33 weeks	1 (2.4)	

^a^ Number of patients where the information is available, ^b^ complicated spontaneous vaginal delivery (SVD) e.g. nuchal cord, breech, shoulder dystocia, forceps or vacuum extraction, C/S: Caesarian section

**Table 4:** Reason(s) for the initial clinical assessment of NF1 patients (N = 50)

Clinical features	Number of patients (%)
Café-au-lait macules	42 (84)
Positive family history of NF1	13 (26)
Developmental delay	5 (10)
Neurofibroma(s)	3 (6)
Failure to thrive	3 (6)
Bony abnormality	2 (4)
Hypotonia	2 (4)
Seizures	2 (4)
Change in vision	2 (4)
Incidental findings on imaging	2 (4)
Tumor	1 (2)
Learning disabilities	1 (2)
Behavioural concerns	1 (2)

**Table 5:** Clinical features in patients with NF1

	Number of patients (%)	^a^ Total number of patients
Learning disability	25 (65.8)	38
Developmental delay	20 (43.5)	46
Speech/language	10 (21.7)	
Global	9 (19.6)	
Gross motor	1 (2.2)	
Headache	17 (34)	^b^ 50
ADHD	14 (28)	^b^ 50
Seizures	8 (16.3)	49
Hypertension	2 (4.8)	42
Neurofibromas	27 (71.1)	38
Excision and/ or debulking of neurofibromas	7 of 27	
Other tumors	28 (59.6)	47
Tumor resection	8 of 28	
Type of other tumors		
Optic pathways glioma	21 (44.7)	
Sarcoma	4 (8.5)	
Astrocytoma	4 (8.5)	
Hemangioma	2 (4.3)	
Glioma (excluding optic pathways)	1 (2.1)	
Craniopharyngioma	1 (2.1)	
Wilm’s tumor	1 (2.1)	
Ovarian renin-secreting tumor	1 (2.1)	
Bony abnormalities	17 (51.5)	33
Scoliosis	12 (36.4)	
Other	4 (12.1)	
Sphenoid bone dysplasia	1 (3.3)	
Family history of NF1		50
Absent	26 (52)	
First-degree relative	10 (20)	
First and second-degree relative	11 (22)	
First, second and third-degree relative	3 (6)	
Café-au-lait macules	50 (100)	50
Axillary and/ or inguinal freckling	40 (88.89)	45
Axillary only	18 (40)	
Inguinal only	2 (4.4)	
Both axillary and inguinal	20 (44.4)	
Decreased visual acuity	9 (19.1)	47
Abnormal visual fields	12 (38.7)	31
Lisch nodules	17 (47.2)	36
Optic disc pallor	18 (40.9)	44
Abnormal pupillary response	5 (12.2)	41
Strabismus	10 (34.5)	29
Nystagmus	4 (25)	16
Decreased tone	9 (23.1)	39
Increased tone	3 (7.7)	39
Decreased strength	6 (15.4)	39
Increased upper/ lower limb reflexes	3 (7.9)/ 8 (21.1)	38
Decreased upper/ lower limb reflexes	4 (10.5)/ 2 (5.3)	38
Clonus	2 (16.7)	12
Babinski sign	3 (8.6)	35
Wheelchair use or other assistive devices	3 (6.8)	44
Clumsy gait and/ or difficulty with fine motor coordination	20 (40)	50

^a^ Number of patients where the information is available, ^b^ the number of patients without headache and attention deficit hyperactivity disorder (ADHD) was not reported; therefore, the percentage represents the least proportion of patients with headaches and ADHD in our cohort

### Neuroimaging

A few brain MRI scans during the early part of the study period were not available for review. Fifty baseline (or the first available) brain MRI scans were reviewed. In addition, if any FASI displayed atypical features then all subsequent available MRI scans were reviewed. Mean age of the patients on the first MRI was 7.8 years (*N* = 50, SD: 4.5 years, median: 7.3 years, minimum: 1.2, maximum: 18.2 years), where SD is the standard deviation. Table 6 shows the number of patients with FASI in different brain locations on the first MRI available. Total FASI count is also displayed. FASI was most commonly seen in the brainstem and basal ganglia in addition to the cerebellum. One patient had two basal ganglia FASI on the first scan but developed FASI in the middle cerebellar peduncle on the second MRI. The rest of the patients had cerebellar FASI on the first MRI. A summary of the distribution pattern of FASI is displayed in Table 7. In many patients, FASI was present concurrently in multiple brain locations.

**Table 6:** The number (no.) of patients with focal abnormal signal intensities (FASI) and the total number of FASI in different brain locations at baseline MRI in all patients

No. of patients	Mean age at MRI (95% CI) in years	The no. of patients with FASI per brain region:					
		Cerebellum	Brainstem	Thalamus	Hypothalamus	Basal ganglia	Cerebrum
50	7.8 (6.5–9.1)	49	31	14	3	36	17
^a^ Median no. of FASI (min.-max.) per patient	Total no. of FASI in all patients	The total no. (%) of FASI in all patients per brain region:					
		Cerebellum	Brainstem	Thalamus & hypothalamus		Basal ganglia	Cerebrum
7 (2–17)	339	136 (40.1)	82 (24.2)	28 (8.3)		65 (19.2)	^a^28 (8.3)

^a^ One patient with the numerous cerebral lesions is excluded, *min*. minimum, *max*. maximum

**Table 7:** The distribution pattern of focal abnormal signal intensities (FASI) on baseline MRI in all 50 patients

Brain location	Number of patient(s)
Cerebellum, brainstem, and (thalamus or basal ganglia)	17
Cerebellum, brainstem, cerebrum, and (thalamus or basal ganglia)	9
Cerebellum and basal ganglia	4
Cerebellum only	3
Cerebellum and cerebrum	3
Cerebellum, cerebrum, and (thalamus or basal ganglia)	3
Cerebellum and brainstem	2
Cerebellum, thalamus, and basal ganglia	2
Cerebellum, brainstem, and cerebrum	2
Cerebellum and thalamus	1
Cerebellum and hypothalamus	1
Cerebellum, basal ganglia, and hypothalamus	1
Cerebellum, brainstem, basal ganglia, and hypothalamus	1
Basal ganglia only initially then middle cerebellar peduncle involvement subsequently	1

FASI was first seen in only four patients below the age of 2 years (8% of all patients). The youngest was 14 months old. In those four patients FASI was present in one or more other brain locations (i.e., basal ganglia, thalamus, brainstem, and cerebrum) in addition to the cerebellum.

A single patient (#54) had widespread numerous FASI in the cortex and subcortical white matter of both cerebral hemispheres. To minimize extreme skewness of the data, we excluded this patient from the analysis involving cerebral FASI counts.

### The longest diameter of FASI

The longest diameter of FASI irrespective of its brain location among our patients in the first scan had a median length of 1.6 cm (*N* = 49, minimum: 0.8, maximum: 5.6 cm). In one patient the image was degraded by motion artifact hence no measurement was done. FASI with the longest diameter were mostly located in the cerebellum on the first MRI (in 22 of 49 patients). In several instances FASI with the longest diameter, especially those > 2.5 cm, was caused by several confluent FASI that could not each be measured individually and separately.

### Atypical features of FASI - mass effect and contrast enhancement

**Fig. 1:**
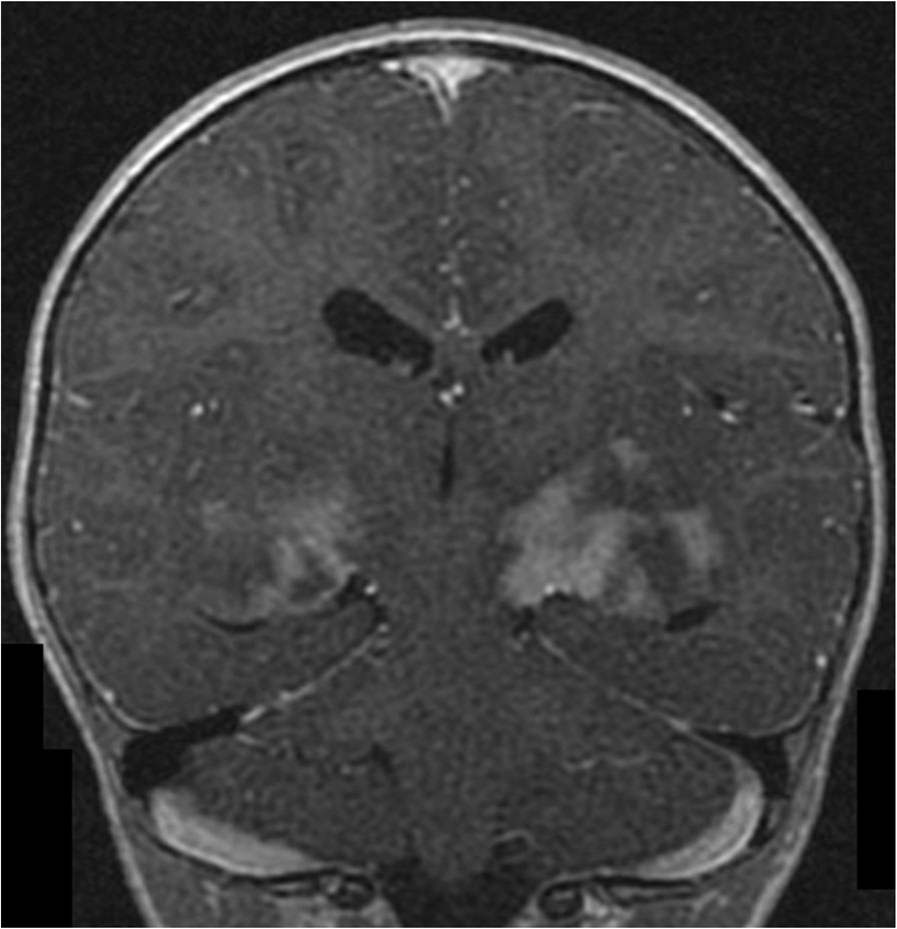
A coronal T1-weighted brain MRI of a 22 months old female with NF1 is shown. There is heterogeneous contrast enhancement involving the basal ganglia and internal capsule bilaterally

**Fig. 2:**
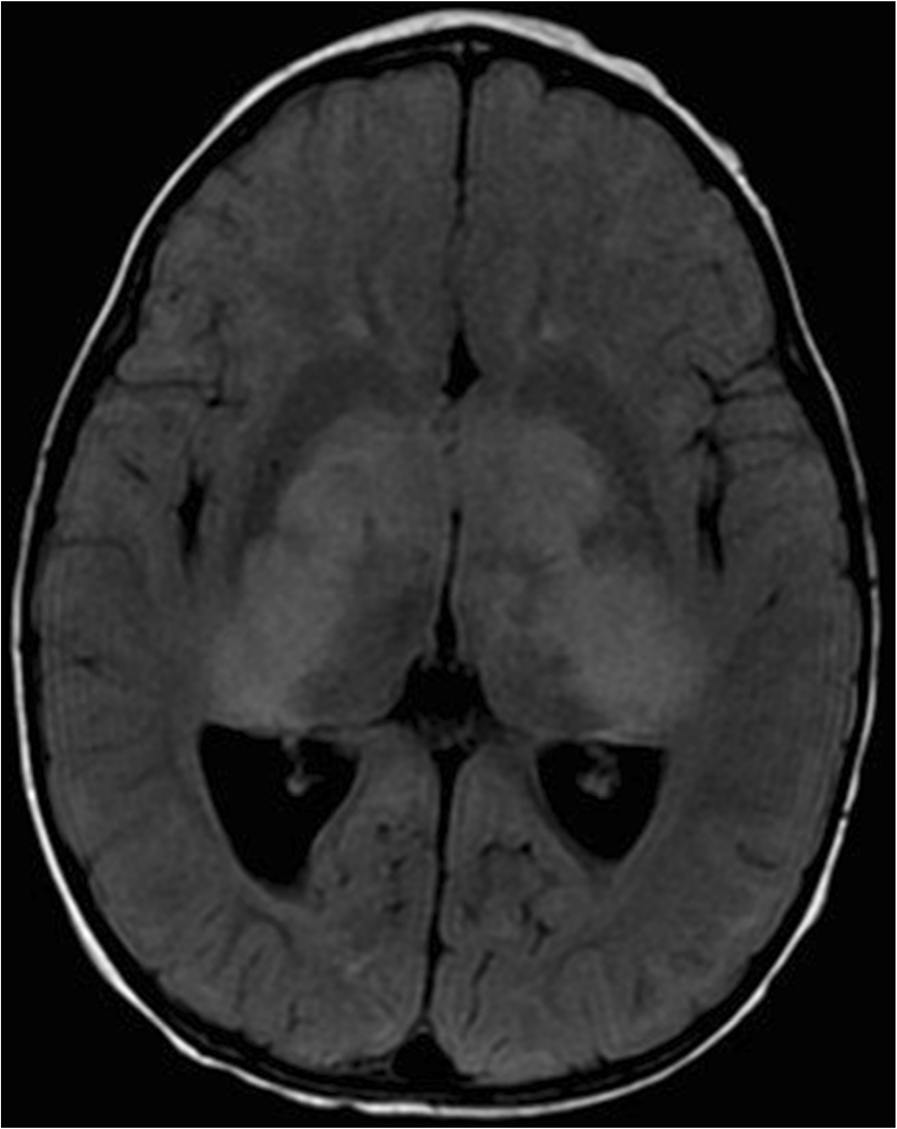
An axial brain MRI of a 22 months old female with NF1 is shown. There are bilateral extensive confluent areas of FLAIR signal abnormalities with increased signal intensity involving the internal capsule bilaterally, basal ganglia, thalami, hypothalamus, and cerebral peduncles. There is mass effect on the posterior horns of the lateral ventricles and on the third ventricle causing dilatation of the posterior horns

**Fig. 3:**
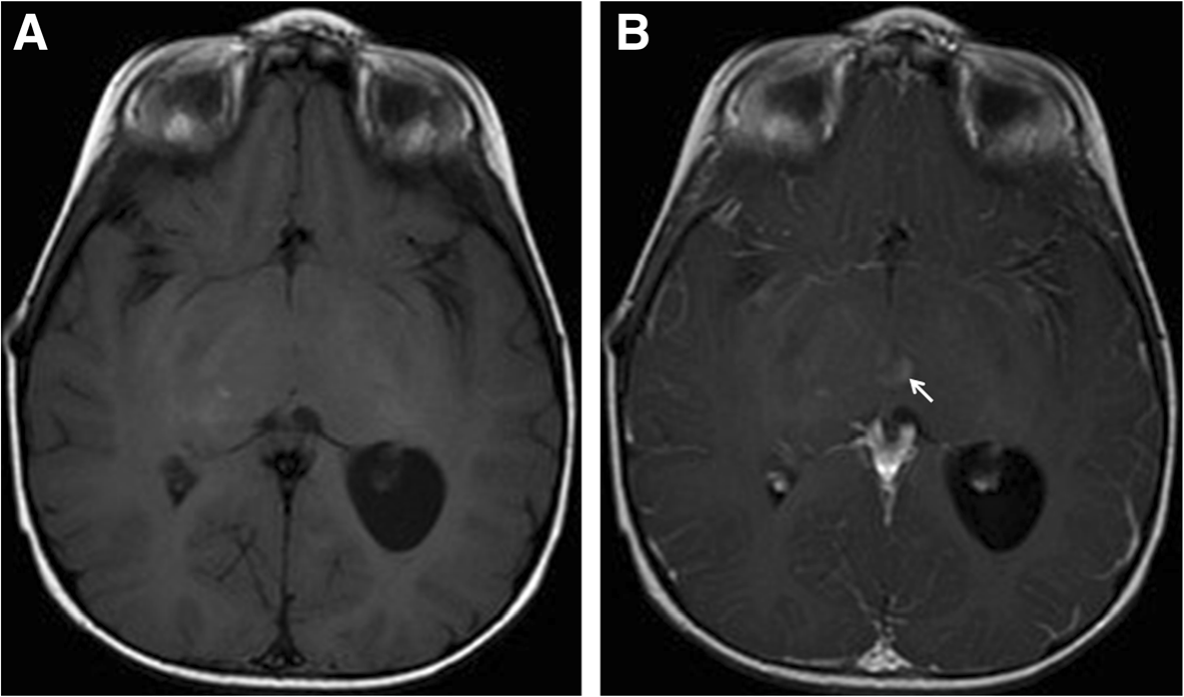
An axial T1-weighted MRI in a two-year old male with NF1 without contrast (A) and with contrast (B) showing an enhancing lesion within the hypothalamus (arrow)

Table 8 shows details of patients with FASI that displayed mass effect, contrast enhancement, or both, and their outcome. Figures 1, 2 and 3 show examples of these atypical features. Mass effect was infrequently seen. Of the 50 patients, mass effect from FASI was noted in 7 (14%) at any scan. Of those 7 patients, the mass effect resolved on subsequent scans in 5 (patients #8, 13, 20, 33, 41 in Table 8), while one patient showed persistence of the mass effect (patient #44 in Table 8). In one patient mass effect was only present on their last scan when FASI became malignant (patient #50 in Table 8).

**Table 8:** Details of patients with NF1 showing mass effect and/or enhancement of focal abnormal signal intensities (FASI)

Patient study number	Age at finding in years (y)	Location	FASI diameter(cm)	Mass effect	Enhancement	FASI outcome
7	17.7	L cerebellum, R basal ganglia	1.2, 0.8	–	+	Remained stable in size with persisting enhancement on a repeat scan 1.3y later. Died at age 28.1y from a pelvic sarcoma
8	12.8	L cerebellum	3.7	+ initially only	+	Enlarged to 4 cm at age 13.1y then shrunk to 2.8 cm with resolution of mass effect and enhancement at age 13.5y. Disappeared at 21.3y
	12.8	R cerebellum	2	+	+	Enlarged to 2.7 cm. Partial excision at age 13y (benign ganglioma). Remnant enhanced then resolved at age 21.8y. Died at age 23.4y from a malignant nerve sheath tumor
13	2.8	L cerebellum	1.2	+	–	Decreased to 0.8 cm with resolution of mass effect at age 5.5y. Disappeared at age 7.6y
	2.8	R cerebellum	1	–	+	Decreased to 0.5 cm with resolution of enhancement at age 5.5y. Disappeared at age 7.6y
	7	R cerebellum	0.8	+	+	Resolved at age 11.2y
20	1.9	L thalamus	1.6	+	–	Resolved at age 2.3y
33	3	R periventricular region	5.6 (few confluent FASI)	+ initially only	+ at 4.6y	Diameter was 4 cm and enhancement persisted on last scan at age 16y
	3	Midbrain	1.6	–	+	Resolved at age 4y
41	11.7	Pons/ L superior cerebellar peduncle	1.6	+	+	Enlarged to 2.2 cm at age 17.2y then shrunk to 1.3 cm with resolution of mass effect at age 19.9y. Enhancement persisted on last scan at age 19.9y
	14.2	L cerebellum	0.8	–	+ at 18.8y	Enlarged to 2 cm and developed into a cystic lesion with a solid component. Fully resected and proven to be a low grade pilocytic astrocytoma
44	1.8	Bilateral basal ganglia	4.9 each	+	+	Shrunk to 2.5, 2.7 cm at age 2.3y then enlarged to 3.9, 5 cm on last scan at age 3.8y. Both mass effect and enhancement persisted
	1.8	L fornix	1.4	+ initially only	–	Enlarged to 2.5 cm at age 2.3y and to 2.7 cm on last scan at age 3.8y
	1.8	Bilateral cerebral peduncles	1.7, 1.4	+ initially only	+	Size remained stable and enhancement persisted on last scan at age 3.8y
50	18.1	R periventricular region	2	+ at 29.3y	+ at 27.4y	Enlarged to 6.7 cm then showed necrosis at age 28.3y, followed by further enlargement to 10.5 cm with edema and mass effect at age 29.3y. Resected and proven to be an anaplastic astrocytoma
	28	Corpus callosum	1.7	–	+	Enlarged to 2.4 cm at age 28.5y then shrunk to 1.9 cm on last scan at age 29.3y with a persisting enhancement
54	3.7	L subcortical white matter	0.5	–	+ at 5.3y	Enlarged to 1.4 cm at age 5.3y then shrunk to 1 cm at age 5.8y. Remained the same size with persistent enhancement on last scan at age 9.2y

*L* left, *R* right, − absent, + present

Contrast enhancement of FASI was also seen infrequently. Of the 50 patients, 2 only had one MRI and contrast was not given. Another 4 patients had no contrast given in only one of their scans. Eight patients showed contrast enhancement at any scan. Of those 8 patients, 2 showed resolution of the contrast enhancement (patients # 8 and 13 in Table 8) and 6 showed persistence of the contrast enhancement (including one patient who was thought to have had only a single scan but a repeat scan during the study period was located later [patient #7 in Table 8]).

### Location of FASI and age at symptom onset/ first clinic visit

There was a significant relationship between the occurrence of FASI in the basal ganglia and age of the patients at symptoms onset. FASI in the basal ganglia occurred at a significantly later age (2 years or older) in comparison with the younger age groups (0–0.9 and 1–1.9 years) at symptom onset (*p* = 0.043). On the other hand, there was a trend for the hypothalamus to be involved in the youngest patients (age 0–0.9 year) in comparison with older patients i.e., 1–1.9, 2–4.9, and 5 years or older at their first clinic visit (*p* = 0.055).

### The relationship between the clinical features and FASI location(s) and total number of FASI

Learning disabilities were significantly less likely in the presence of FASI in the thalamus (*p* = 0.033). A similar trend was also found for developmental delay and thalamic FASI (*p* = 0.08). On the other hand, a trend between developmental delay and the presence of FASI in the hypothalamus was present (*p* = 0.075). Otherwise, no other relationship between FASI location and other clinical symptoms was seen. In addition, none of the clinical symptoms showed any significant relationship with various combinations of FASI in different brain locations.

As for clinical signs, univariate analysis revealed that strabismus was significantly more likely to be associated with basal ganglia FASI (*p* = 0.009). In addition, abnormal visual fields were significantly less likely with thalamic FASI (*p* = 0.044), while abnormal visual fields and fundi exam were significantly more likely with cerebral FASI (*p* = 0.035 and *p* = 0.005, respectively). Multiple logistic regression of cerebral involvement with symptoms and signs showed that the odds of cerebral involvement in children with abnormal fundi was 7.46 (95% CI, 1.4–40, *p* = 0.019) times higher than normal fundi after adjusting all other symptoms and signs variables.

When various combinations of brain locations of FASI were correlated with clinical signs, there was a trend for abnormal visual fields not to be associated with the presence of FASI in all of the following locations: cerebellum, thalamus or basal ganglia, and brainstem (*p* = 0.079), while abnormal fundi were significantly associated with widespread involvement of FASI in all brain regions (*p* = 0.009). However, on multivariate analysis where funduscopy and visual fields were paired, the latter association was no longer significant.

When the clinical features were correlated with the total number of FASI on baseline MRI, significant associations were found in patients with attention deficient hyperactivity disorder (ADHD) (*p* = 0.01) and in patients with impaired visual acuity (*p* = 0.019) after adjusting for all other clinical features. The expected number of FASI at baseline scan for patients with ADHD was 39.3% lower than for those without ADHD after adjusting for all other variables (i.e., there was a negative association). In addition, the expected number of FASI at baseline scan for patients with impaired visual acuity was 60.1% higher than for those with normal visual acuity after adjusting for all other variables (i.e., there was a positive association).

There was only an overall trend (*p* = 0.076) for a positive history of NF1 *in any family member* to be associated with the total number of FASI on baseline MRI. However, this association became significant if a first degree relative rather than a more distant relative had NF1 (*p* = 0.029). The expected number of FASI at baseline scan for patients with a first degree relative with NF1 was 56.1% higher than for those with no family history of NF1 after adjusting for all other variables.

### Optic pathways gliomas and their relationship with visual signs, FASI locations, and FASI counts

Of the 50 patients, 18 had optic pathways gliomas on their initial MRI scan. Abnormalities in the visual acuity, visual fields, and funduscopy exam were significantly associated with optic pathways gliomas on baseline brain MRI (*p* = 0.0006, *p* = 0.011, and *p* < 0.0001, respectively).

There was a significant association between the presence of optic pathways gliomas on baseline MRI and the number of patients with FASI in the cerebrum but not in other brain locations. Cerebral FASI was present significantly more commonly in patients with optic pathways gliomas, than in patients without optic pathways gliomas on baseline MRI (58.8% versus 41.2%, *p* = 0.019). Furthermore, the mean number of FASI in the supratentorial region and in the cerebrum (but not in other brain locations) were each significantly higher in patients with optic pathways gliomas (*N* = 18), than in patients without optic pathways gliomas (*N* = 31) on baseline MRI (mean FASI count = 4.46 versus 2.93 for the supratentorial region, *p* = 0.008; mean FASI count = 1.11 versus 0.27 for the cerebrum, *p* = 0.007).

### The association between maternal age at conception and the number of FASI and clinical features

There was a significant relationship between maternal age at conception (available in 41 patients) and the number of infratentorial FASI on baseline MRI (*p* = 0.029). With every one-year increase in maternal age at conception, the expected number of infratentorial FASI at baseline MRI was 2.75% lower. Similarly, there was a significant relationship between maternal age at conception and the number of brainstem FASI on baseline MRI (p = 0.029). With every one-year increase in maternal age at conception, the expected number of brainstem FASI at baseline MRI was 3.98% lower. There was only a similar trend between maternal age at conception and the number of basal ganglia/ internal capsule FASI on baseline MRI (*p* = 0.067).

Two trends were found for associations between decreasing maternal age at conception and the increased presence of learning disabilities (*p* = 0.065) and ADHD (*p* = 0.081) in children with NF1.

## Discussion

Our investigation focuses on a subgroup of patients with NF1 and cerebellar FASI on their MRI scans during childhood. We selected them a priori to investigate cerebellar FASI and their clinical impact. Recently, there has been much interest in the expanding roles of the cerebellum and its important role in non-motor in addition to motor tasks [18]. Our patients represent what is commonly seen in clinical practice since many patients with NF1 have FASI [12, 13, 20], and 23.5–84% of these patients also have FASI in the cerebellum/ brainstem [13, 14, 17, 21, 22].

The diagnosis of NF1 was made early in our cohort. A positive family history of NF1, reported commonly in our patients (48%), and similar to other studies (e.g. 35% [17], and 54.8% [23],), and the frequent presence of café-au-lait macules likely prompted an early referral and diagnosis. Tumors (especially neurofibromas), developmental delay, and learning disabilities occurred commonly in our patients, while headaches, ADHD, and seizures were less frequent clinical features. Their prevalence is similar to prior reports [24, 25]. The prevalence of other features including café-au-lait spots, axillary and inguinal freckling, Lisch nodules, and sphenoid dysplasia were also similar to other studies [17, 26].

Visual signs e.g. decreased visual acuity, abnormal visual fields, and optic discs pallor, detected commonly on examination, were important markers associated with optic pathways gliomas. Indeed, optic pathways gliomas occurred commonly and were mostly present on the initial MRI scans in our study. These anticipated findings have been reported previously and are consistent with impaired optic nerve function in some patients with NF1 and optic pathways gliomas [27].

There was high prevalence of optic pathways gliomas (44.7%) in our cohort in comparison with several studies (4–25%) [17, 24, 25, 27]. A large study in patients with NF1 reported a significant association between the presence of FASI and optic pathways gliomas (odds ratio: 2.1, 95% CI: 1.2–3.6) [26], which may account for our finding since all our patients had FASI. However, in a study of 31 children with NF1 who had brain MRI, there was no correlation between FASI (present in 27 patients) and optic pathways gliomas, which similar to our study was noted in 14 (45%) of the patients [23]. In another study investigating the natural history of FASI, optic pathways gliomas were present in 33% of 46 patients with NF1 [13]. The higher prevalence of optic nerve gliomas in our study may also be due to selection bias, since such patients are more likely to be seen in a tertiary hospital and have neuroimaging. Similar referral and selection bias may explain the higher proportion of our patients with NF1 who developed other neoplasms (24%) than the prevalence of neoplasms reported in other studies (4–10.7%) [24, 25].

On the other hand, cerebellar motor signs were absent in our cohort, consistent with a normal cerebellar motor function despite the presence of cerebellar FASI. While clumsiness, lower manual dexterity score, and impaired fine motor skills or coordination has been reported in NF1 patients, the occurrence of frank cerebellar ataxia has not [11, 12, 20, 28, 29], unless other complicating factors are present, such as an expanding posterior fossa or spinal cord tumor or high cervical cord lesions compressing the cord [1, 6].

The nature of FASI is unclear but may be related to increased fluid within the myelin associated with hyperplastic or dysplastic glial proliferation as suggested in a study using newer MRI techniques, e.g. multi-exponential T2 relaxation and diffusion MRI including diffusion tensor imaging and diffusion kurtosis imaging [30]. Developmental anomalies such as hamartomas, dysplasias, and heterotopia would not be anticipated to produce reversible signal abnormalities [13, 21].

Studies documenting the neuroimaging-pathological correlation of FASI are rare. In one study, three pediatric patients with NF1 had autopsy which showed that the pathological correlation of FASI were areas of fluid-filled vacuolar or spongiotic change [31]. There was no evidence of demyelination, inflammation, gliosis, stainable material, or axonal damage. Diffusion tensor imaging on 50 children with NF1 showed higher ADC values not only in FASI but also in normal appearing white matter in patients in comparison to control patients without NF1, reflecting an increase in the magnitude of water molecules diffusion and microstructural damage. The specifically higher diffusivity (measured in eigenvalues) found only in FASI was consistent with microstructural abnormalities caused by decreased axonal packing, intramyelinic edema, vacuolation, or fluid accumulation [32]. Fractional anisotropy (FA) was significantly lower in FASI located in the cerebellar white matter only. In another study of 27 children and young adults with NF1, FA decreased in the cerebellum, thalamus, and basal ganglia but only in NF1 patients whose FASI decreased in number and volume in those regions, suggesting persistent microstructural damage even when FASI disappear [15]. Similar conclusions were reported in 15 children with NF1, where ADC values were high in normal appearing brain and highest in FASI in comparison to healthy controls. The ADC values in the locations where FASI regressed in some patients, were higher than normal appearing brain in these patients, suggesting that macroscopic resolution of FASI on MRI does not necessarily lead to the full resolution of the microstructural abnormalities [33].

Brain MRI scans in our children with cerebellar FASI showed that FASI rarely occur in isolation and most commonly occur in multiple brain regions in addition to the cerebellum, especially the brainstem and basal ganglia as noted before [15, 17, 19, 22], i.e. FASI showed no predilection to a specific brain region in our patients.

There was a suggestion that age at symptom onset or first clinic visit may correlate with where FASI develops, since FASI that developed in the basal ganglia occurred in patients who were relatively older at age of symptom onset. There was a tendency to develop hypothalamic FASI in patients who were relatively younger at the age of their first clinic visit.

FASI developed below the age of 2 years in different brain locations in a few of our patients, which is not considered to be typical by some authors [21], and especially as myelination is incomplete [17, 33]. The total or regional number of FASI was independent of the age of symptom onset or age at first clinic visit, consistent with their asymptomatic development.

Prior studies have shown that cognitive deficits were more common in patients with NF1 and thalamic FASI [11, 12], especially if the lesions are well circumscribed [34]. However, the association is controversial since other studies showed no such association with executive/ cognitive function in terms of the presence, number, size, or location of FASI [20, 22, 23]. In our investigation, learning disabilities, reported in 30–65% of children with NF1 [20], and developmental delay (for which there was only a trend), occurred significantly less commonly in patients with NF1 and thalamic FASI. However, our patients did not have formal neuropsychological testing. In addition, our study design is different from the aforementioned studies [11, 12], since all our patients also had cerebellar FASI as part of the inclusion criteria. We only found a trend for developmental delay to occur more commonly in patients with hypothalamic FASI. Furthermore, there was no significant relationship between any of the clinical symptoms and several combinations of brain locations where FASI were present. A longitudinal study of cognitive function and FASI with long term follow up showed that if patients with NF1 had FASI then their IQ is less during childhood but their IQ increases to average values when FASI disappears in early adulthood [35]. One study reported that patients with cerebellar FASI had significantly lower full scale IQ and verbal IQ scores in comparison with NF1 patients without cerebellar FASI [10]. We were not able to investigate this finding since our patients did not undergo detailed cognitive testing and all had cerebellar FASI. However, several of our patients did not have developmental delay or learning disabilities, suggesting that having cerebellar FASI per se does not necessarily leads to impaired development or learning.

As for clinical signs, visual fields and especially funduscopic abnormalities were associated more commonly with patients who had cerebral FASI. This can be explained through confounding with the presence of optic pathways gliomas on baseline MRI, since cerebral FASI was seen significantly more commonly in patients whose baseline MRI also showed optic pathways gliomas. Strabismus was significantly associated with the presence of FASI in the basal ganglia. We found no studies that directly link strabismus with disorders of the basal ganglia. In one study of 213 children with cerebral palsy, strabismus was reported in 3 of 15 children with dyskinetic cerebral palsy. Abnormalities in the basal ganglia are implicated in dyskinetic cerebral palsy [36]. Our finding may have arisen by chance. Further corroboration is needed before any definitive conclusions can be made.

The total number of FASI at baseline MRI was significantly less in patients with ADHD and more if a first degree relative had NF1 and also more in patients with decreased visual acuity. This latter association may have arisen through the occurrence of optic pathways gliomas, which themselves were associated with higher mean numbers of FASI in the supratentorial regions and more specifically in the cerebrum. The former two associations i.e. ADHD and family history of NF1, may be caused by genetic factors/ predisposition but this is speculative. FASI was not associated with the presence of ADHD in 76 NF1 patients, who were preselected and did not have epilepsy or optic nerve glioma [34].

The relationships between decreasing maternal age at conception and the increasing number of FASI at baseline (statistically significant) or the increasing presence of learning disabilities and ADHD (statistical trends) are intriguing; albeit, small in magnitude. They raise the question on whether decreasing maternal age at conception per se or through confounding factors such as socioeconomic status or educational achievement [37], somehow influences the development of FASI, especially in the brainstem region, or adversely affects learning and behavior. It will need further validation and their clinical significance in patients with NF1 is unclear at this point since adverse health outcomes (including cognitive disability and ADHD) and maternal age at conception, whether in younger or older mothers, occurs independently of NF1 [37–39]. In one study in patients with NF1, learning disability was not associated with maternal age at conception. The presence of ADHD in the cohort was not reported [40].

A few FASI showed contrast enhancement, mass effect, or both. Such findings are reported to occur rarely [13, 14, 41, 42], but are not considered to be typical of FASI in NF1 [13, 21]. They are concerning for the development of neoplasms [5]. Mass effect was seen mostly initially and tended to resolve in our patients. On the other hand, contrast enhancement tended to persist on repeat MRI scans. In a few of our patients contrast enhancement was still present 16 months - 11 years of follow up. On only one occasion, a single FASI located in the periventricular region developed a mass effect later and became malignant (patient #50 in Table 8). In two other patients, a cerebellar FASI enlarged and displayed contrast enhancement in each patient. Their biopsies revealed a benign ganglioma and a low grade pilocytic astrocytoma (patients #8 and 41 in Table 8). Tumors in the cerebellum and cerebral hemispheres are uncommon in patients with NF1 [7], and most are benign [6, 8]. However, they occur more commonly in patients with FASI [26]. In 5 of 46 patients with NF1, FASI enlarged, showed mass effect, and enhanced [13]. Biopsy was performed in one other patient, whose brain MRI with contrast was normal initially but who then developed several FASI and also a large enhancing mass in the splenium of the corpus callosum. When the latter increased further in size, a biopsy revealed grade II astrocytoma. The rest of the patients with enhancing FASI were observed and no further follow up information on their outcome was given. The authors advised a wait-and-watch approach in such otherwise concerning cases, since current medical wisdom dictates that mass effect and enhancement of a lesion are suggestive of a tumor but at the same time tumor regression without treatment is recognized in NF1 [2, 5, 13]. In summary, enlargement in the size of FASI is well documented. However, infrequently a few FASI tend to also develop a transient mass effect and enduring contrast enhancement with time [14, 41, 42]. Sometimes this enduring contrast enhancement resolves [41], and only rarely does malignancy develops. Therefore, we support the clinical practice of repeating brain MRI in patients with NF1 who develop new clinical symptoms or signs (e.g. those suggestive of raised intracranial pressure or hemiparesis [43],) or in patients, whose FASI show mass effect and enhancement, since very rarely they can become malignant. If FASI becomes cystic, as we found in one of our patients, a tumor is highly likely [5, 43]. It is still unknown whether the same neuropathology occurs in the lesions that enhance or show mass effect in comparisons with FASI that do not enhance or show mass effect [13].

Limitations of our study include incomplete ascertainment of patients, missing data, variable durations of follow up, variable ages at which the MRI scans were done, non-uniform MRI magnetic strength and protocols used in these patients, difficulties associated with measuring the diameter of ill-defined FASI, and parental report of learning disabilities were not formally verified through comprehensive cognitive assessment.

## Conclusions

Patients with NF1 and cerebellar FASI do not have cerebellar motor symptoms or signs. If ataxia develops then neuroimaging is indicated. Patients with NF1 and cerebellar FASI may or may not have learning disabilities or developmental delay and therefore, cerebellar FASI cannot be implicated directly as a causative factor for non-motor cerebellar symptoms. FASI occurred in several brain locations and were rarely confined to the cerebellum in our preselected patients. FASI has the potential, though uncommonly, to show mass effect and/ or contrast enhancement. Rarely, such features herald a malignant change.

The total number of FASI at baseline MRI was significantly less in patients with ADHD and more if a first degree relative had NF1 and also more in patients with decreased visual acuity. The latter association is likely caused by the presence of optic pathways gliomas in our cohort. The influence of maternal age of conception on the development of FASI or its association with a few clinical features requires further study.

## Abbreviations

ADC: Apparent diffusion coefficients
ADHD: Attention deficient hyperactivity disorder
FA: Fractional anisotropy
FASI: Focal abnormal signal intensities
FLAIR: Fluid-attenuated inversion recovery images
FSPGR: Fast spoiled gradient echo images
NF1: Neurofibromatosis type 1
